# *Alcaligenes xylosoxidans* Bloodstream Infections in Outpatient Oncology Office

**DOI:** 10.3201/eid1407.070894

**Published:** 2008-07

**Authors:** Moon J. Kim, Elizabeth Bancroft, Eleanor Lehnkering, Rodney M. Donlan, Laurene Mascola

**Affiliations:** * Los Angeles County Department of Public Health, Los Angeles, California, USA; † Centers for Disease Control and Prevention, Atlanta, Georgia, USA

**Keywords:** Alcaligenes xylosoxidans, outbreak, outpatient, central venous catheter, bacteremia, research

## Abstract

In 2002, we investigated a cluster of patients with *Alcaligenes xylosoxidans* bloodstream infections by conducting a matched case–control study and a prospective study. Pulsed-field gel electrophoresis (PFGE) was performed on blood culture isolates, and 1 explanted central venous catheter (CVC) was tested for biofilm. We identified 12 cases of *A. xylosoxidans* bloodstream infection. Case-patients were more likely than controls to have had a CVC (7/7 [100%] vs 4/47 [8.7%], respectively; p<0.0001). Ten case isolates were indistinguishable by PFGE analysis, and *A. xylosoxidans* biofilm from the CVC matched the outbreak strain. We observed multiple breaches in infection control, which may have caused contamination of multidose vials used to flush the CVCs. Our study links *A. xylosoxidans* with CVC biofilm and highlights areas for regulation and oversight in outpatient settings.

## Abstract 2

Gaps in infection control led to biofilm production in central venous catheters and resultant bloodstream infection.

*Alcaligenes xylosoxidans*, also known as *Achromobacter xylosoxidans,* is a gram-negative, water-borne organism that causes healthcare-associated infections (*1*–*7*) and bacteremia in immunocompromised patients with indwelling catheters (*6*–*11*); it can also contaminate liquids (*2*,*5*,*12*–*14*). *A. xylosoxidans* is found in soil and water and grows in saline (*15*,*16*). On January 16, 2002, the Acute Communicable Disease Control (ACDC) program of the Los Angeles County Department of Public Health was notified by a local hospital epidemiologist about a cluster of patients with *A. xylosoxidans* bloodstream infections (*17*). The patients had been admitted to Hospital A over a period of 2 months.

To confirm the presence of an outbreak, ACDC conducted a telephone survey, which asked the microbiology laboratories of Hospital A and 4 surrounding hospitals for a list of all patients who had had positive blood cultures for *A. xylosoxidans* in the past 3 months. One laboratory identified 3 such patients, and Hospital A laboratory identified 7; all 9 patients (1 case-patient had positive blood cultures reported from both laboratories) were associated with a single outpatient oncology office, Office B. The other 3 hospitals reported that they had not identified any *A. xylosoxidans* bloodstream infections in the past 3 months. To identify the source of the outbreak and risk factors for infection and to implement control measures, ACDC then initiated an outbreak investigation.

## Methods

The outbreak investigation focused on Office B. To identify all patients associated with Office B who had had a positive *A. xylosoxidans* culture, we requested a review of medical records and laboratory reports.

### Matched Case–Control Study

A matched case–control study was performed to determine risk factors for infection. A case-patient was defined as a patient of Office B who had a positive *A. xylosoxidans* blood culture from November 2001 through January 2002. Controls were defined as patients who had no symptoms or signs of bloodstream infection (fever, chills, rigors, myalgias, nausea, vomiting, weakness, or hypotension). For each case-patient, 5–7 controls were randomly selected and matched by the closest date of their visits to Office B to the case-patient’s date of visit. Variables included age, sex, underlying diagnosis, intravenous medications received, peripheral white blood cell counts, presence and type of central venous catheter (CVC), clinic visits, hospitalization dates, symptoms, and types of invasive procedures. Data were collected on standardized forms and analyzed by using Epi Info 2000 version 1.1.2 (Centers for Disease Control and Prevention [CDC], Atlanta, GA, USA); odds ratios were used to estimate risk, *t* tests were performed for continuous variables, and p<0.05 indicated statistical significance.

### Prospective Cohort Study

To identify possible *A. xylosoxidans* bloodstream infection, ACDC conducted prospective blood culture surveillance. On February 15, 2002, all patients with a CVC who had visited Office B since November 2001 were sent a letter requesting them to have a culture performed on blood drawn through the CVC. CVCs were removed from those whose culture results were positive.

### Environmental Investigation

On January 17, 2002, numerous open containers (multidose 30-mL vials of heparin; 100-mL and 150-mL bottles of saline; and containers of alcohol, hydrogen peroxide, betadine, and iodine) were collected and sent to the Los Angeles County Public Health Laboratory for analysis. On February 15, 2002, environmental samples and swabs were collected for culture from work surfaces (e.g., countertops, sinks, hoods, kitchens) and from tap water and hands of healthcare workers who accessed CVCs, collected blood, prepared flushes, or administered chemotherapy.

### Infection Control

During January and February 2002, we made several site visits to Office B to observe procedures, review medical records, and interview the office staff. Specifically, we observed procedures for preparation and administration of intravenous medications and asked office staff about level of technical training, experience, and license status.

### Molecular Studies

Blood isolates of *A. xylosoxidans* from case-patients were obtained from Hospital A’s laboratory. For comparison, all *A. xylosoxidans* isolated from patients from Los Angeles County in the past 6 months were obtained from a large local reference laboratory. Pulsed-field gel electrophoresis (PFGE) was performed at the Los Angeles County Public Health Laboratory by using standard methods (*18*) for *Salmonella* spp. with the exception that isolates were digested with *Xba*I and *Spe*I. PFGE pattern comparisons were performed visually by using criteria established by Tenover et al. (*19*).

### Examination of CVC for Biofilm

A CVC (PASport, a single-lumen, 6 French catheter with an under-the-skin titanium port; SIMS Deltec, Inc., St. Paul, MN, USA) was surgically removed from an asymptomatic patient identified in the prospective cohort study. Aseptic methods were used. The distal lumen opening was clamped with a sterile hemostat to retain the liquid within the lumen, and the catheter was placed in a sealed, sterile container and shipped overnight to CDC in Atlanta for processing within 24 hours of collection. At CDC, the CVC was placed into a Class II Biological Safety Cabinet, and a 1-cm segment was removed with a sterile scalpel. This segment was cut lengthwise to expose the lumen, and the individual pieces were placed into 5% glutaraldehyde (Ted Pella, Redding, CA, USA) in 0.67 M cacodylate buffer, pH 6.2, and processed for scanning electron microscopy (*20*). Samples were examined by using a Philips XL 20 Scanning Electron Microscope (FEI Company, a subsidiary of Philips, Hillsboro, OR, USA). The remaining catheter attached to the titanium port was clamped, and the outer surface was cleaned with a 70% alcohol wipe, disinfected, and processed to recover biofilm organisms (*20*). The recovered organisms were plated on trypticase soy agar containing 5% sheep blood (blood agar; Becton, Dickinson, Sparks, MD, USA). Plates were incubated for up to 72 h at 35°C and then examined. Colonies were spread onto blood agar for subculture and identified by using standard clinical microbiologic methods (*21*). Biofilm isolates were also characterized by PFGE (methods described above).

## Results

A total of 12 patients with *A. xylosoxidans* bloodstream infection were found: 9 from the retrospective case–control study and 3 from the prospective study (Table). All 12 were immunocompromised. Their ages ranged from 41 to 79 years (mean 65.8 years), and 10 (83.3%) were female. Case-patients had differing underlying diagnoses and chemotherapy regimens. Case-patients had had fevers, chills, and/or rigors within minutes to days after an infusion through their CVC. Several case-patients had multiple episodes of fever and chills during and immediately after visits to Office B when their CVC was accessed for blood collection, chemotherapy, or routine flushes. For some, these symptoms were attributed to possible side effects of chemotherapy. All case-patients had visited Office B from November 12 through December 18, 2001. Case-patient 1 was hospitalized from October 12 through November 10, 2001, and had visited Office B for daily collection to monitor neutropenia from November 13 through 19, 2001. Patients with *A. xylosoxidans* bloodstream infection were treated with antimicrobial drugs and CVC removal. Available records showed case-patients were treated with piperacillin/tazobactam; 1 case-patient who was allergic to penicillin was treated with aztreonam. One patient died from underlying malignancy (end-stage pancreatic cancer).

**Table:** Characteristics of 12 case-patients with bacteremia caused by Alcaligenes xylosoxidans*

Case-patient no.	Age, y	Sex	Underlying disease	Date of CVC flush	Date of onset, symptoms†	Date of blood collection‡
Case–control study						
1	70	F	Acute myelogenous leukemia	2001 Nov 19	2001 Nov 19, rigors after flush	2001 Nov 19
2	65	F	Breast cancer	2001 Dec 5	2001 Dec 5, no chart record of symptoms after flush, blood culture obtained next day	2001 Dec 6
3	73	F	Colon cancer	2001 Dec 7	2001 Dec 7, fever after flush	2001 Dec 7
4	41	F	Sickle cell disease	2001 Dec 4	2001 Dec 11, myalgias, emesis	2001 Dec 11
5	73	F	Gastric cancer	Unknown	Unknown	2001 Dec 13
6	71	M	Colon cancer	2001 Dec18	2001 Dec 19, fever, chills	2001 Dec 20
7	79	F	Pancreatic cancer	Unknown	Unknown	2001 Dec 30
8	45	F	Breast cancer	2002 Jan 10	2002 Jan 10, no chart record of symptoms after flush, blood culture obtained next day	2001 Jan 10
9	70	F	Non-Hodgkin lymphoma	2002 Jan 10	2002 Jan 10, hypotension after infusion	2002 Jan 11
Prospective study						
10	77	F	Squamous cell cancer of palate	NA	2001 Nov/Dec, intermittent nausea and weakness	2002 Jan 29
11	77	M	Gastric cancer	NA	2001 Nov/Dec, intermittent fever, chills after flushes, not reported to Office B staff	2002 Feb 7
12	49	F	Non-Hodgkin lymphoma	NA	Asymptomatic	2002 Feb 5

*CVC, central venous catheter; NA, not applicable. †Signs and symptoms of bloodstream infection. ‡Collection of blood that had subsequent positive culture result for *A. xylosoxidans*.

### Matched Case–Control Study

Of the 9 case-patients identified, 7 who had clear onset date of bloodstream infection symptoms were selected for the case–control study. Case-patients were younger than controls (mean age 63.5 years [range 41–73 years] and mean age 73.2 years [range 35–89 years], respectively; p = 0.047). Case-patients were significantly more likely to have a CVC than controls. Matched case–control analysis showed that all 7 case-patients versus 4 of 47 control patients had a CVC at the time of illness onset (p<0.0001). The 2 other case-patients not included in the case–control study also had CVCs. Patients with CVCs received heparin and saline flushes before and after the CVC was used for blood collection or infusions. No records documented when each of the Office B nurses accessed the CVCs. Patients without CVCs who needed only blood collection for testing did not receive any flushes; however, those without CVCs who needed blood tests before receiving an infusion received a heparin and saline flush after a peripheral intravenous line was placed. Case-patients and controls did not have statistically significant differences in peripheral leukocyte counts, intravenous medications administered, types of chemotherapy received, or underlying diseases.

### Prospective Cohort Study

In February 2002, 29 patients with CVCs had blood collected for culture. Of the 3 (10%) who had positive culture results for *A. xylosoxidans,* chart review showed that 2 had been intermittently symptomatic (Table).

### Environmental Investigation

Cultures from available open solutions in the oncology office, collected 6 weeks after the initial cluster of *A. xylosoxidans*–positive blood cultures, and environmental cultures did not grow *A. xylosoxidans*. A sample from a sterile saline bottle that was open in the infusion room was positive for *Bacillus circulans*, and a tap water sample was positive for *Moraxella* spp.

### Infection Control Practices and Procedures

Office B had 10 patient examination rooms and a separate, large, open infusion room where several patients could receive chemotherapy. The infusion room contained a hood and sink for preparation of intravenous medication. Of the 4 staff members at Office B who regularly accessed CVCs; inserted peripheral intravenous catheters; collected blood; and prepared or administered chemotherapy, flushes, or intravenous medications, only 1 was a registered nurse who had a California state license. The 3 nonlicensed staff members were reported to have received nursing training in their native country but did not have documented formal training or education. One nurse wore artificial fingernails but had removed them before hand culture samples were collected; thus, the fingernails were unavailable for culture. The following breaches in infection control were noted: intravenous catheters were inserted by persons not wearing gloves; unlabeled, prefilled syringes were stored in the hood; no documentation of hood cleaning was found; open, multidose heparin vials and saline bottles, some undated, were found throughout the facility; nonhygienic material was stored in the chemotherapy medication preparation hood; and failure to wash hands between patients was noted. No pharmacists were employed at Office B. No documentation of staff training and evaluation for chemotherapy preparation or infection control competency was available.

### CVC Biofilm Studies

**Figure 1:**
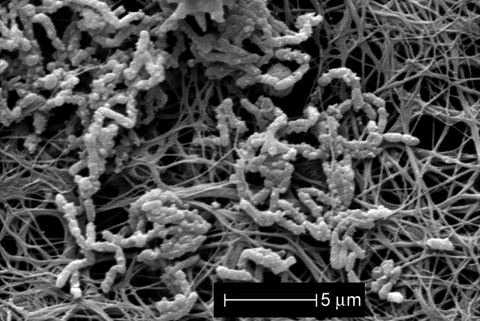
Scanning electron micrograph of lumen of segment of central venous catheter removed from an asymptomatic patient. Biofilm contains rod-shaped bacteria (*Alcaligenes xylosoxidans)* in association with fibrinlike material on the catheter surface.

Scanning electron microscopy of the CVC showed a biofilm that contained rod-shaped bacteria in association with fibrinlike material on the catheter surface (Figure 1). A pure bacterial culture recovered from the CVC lumen was identified as *A. xylosoxidans*.

### Molecular Studies

**Figure 2:**
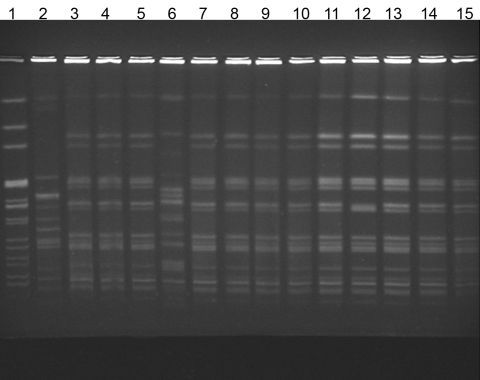
Pulsed-field gel electrophoresis of isolates from patients with *Alcaligenes xylosoxidans* bloodstream infection. Lane 1, laboratory standard; lanes 2 and 6, community strains of *A. xylosoxidans*; lanes 3–5 and 7–13, outbreak strains; lane 14, central venous catheter (CVC) port biofilm outbreak strain; lane 15, CVC port outbreak stain.

*A. xylosoxidans* blood culture isolates from case-patients were indistinguishable by PFGE analysis (Figure 2); in contrast, 3 *A. xylosoxidans* isolates from a local reference laboratory had different PFGE patterns. The isolate from the CVC biofilm matched the *A. xylosoxidans* bloodstream infection outbreak strain.

## Discussion

This large outbreak (N = 12) of *A. xylosoxidans* bloodstream infections was caused by 1 strain, which was also isolated from CVC biofilm. Symptoms of bloodstream infection probably occurred when flushes detached bacteria from the CVC biofilm. The prospective study found that 3 (10%) of 29 patients had *A. xylosoxidans–*positive blood cultures. Our case–control and prospective studies support the association of *A. xylosoxidans* bloodstream infection and CVCs, and our molecular biologic studies confirm A. *xylosoxidans* biofilm of the same outbreak strain on a CVC. *A. xylosoxidans* outbreaks reported to date have been associated with healthcare and contamination of hospital products (*1*,*2*,*5*,*12*–*14*), but none occurred in an outpatient setting.

The cause of this outbreak most likely was the use of contaminated multidose vials of heparin or saline flushes, leading to the formation of an *A. xylosoxidans* biofilm on CVCs. Case-patient 1 had been hospitalized from late October through early November at Hospital A. During November 13–19, 2001, this case-patient had had blood collected and her CVC line flushed numerous times at Office B; on November 19, culture result indicated *A. xylosoxidans* infection, which was successfully treated. We observed multiple breaches in infection control at Office B: use of unlabeled, prefilled syringes, poor hand hygiene, and lack of glove use. The CVC of case-patient 1 may have been flushed by using the same syringe and needle inserted into multidose vials, causing contamination of the vials. Another possible route of contamination is through artificial fingernails. A cluster of *Serratia marcescens* and *A. xylosoxidans* bacteremia cases linked to multidose heparin vials contaminated by a nurse with artificial fingernails has been reported (*22*); however, the artificial fingernails from the nurse at Office B were unavailable for testing. We suspect that multidose vials were contaminated with *A. xylosoxidans* and subsequently used on other patients from November 12 through December 18, 2001, when all case-patients had overlapping visits at Office B and received CVC flushes. A culture from an open, supposedly sterile saline bottle grew *B. circulan*s, which suggests possible breaches in infection control. Multidose heparin and saline vials have been reported as the cause of outbreaks of hepatitis C (*23*,*24*), *S. marcescens* (*25*), and *Pseudomonas aeruginosa* (*26*) infections.

Although the heparin and the saline vials could have been contaminated from case-patient 1 in November, case-patients who subsequently received flushes from these vials may not have become immediately ill with symptoms of bloodstream infection. *A. xylosoxidans* biofilm could have developed on their CVCs and intermittently caused clinical illness when the CVCs were manipulated; i.e., flushing dislodged the biofilm and caused symptomatic bacteremia. Although indwelling catheters are frequently colonized with biofilm shortly after insertion (*27*), colonization does not necessarily lead to infection; bloodstream infection symptoms from an organism in contaminated intravenous solutions have been delayed for as long as 421 days after exposure (*28*). The finding of an asymptomatic patient with a CVC with *A. xylosoxidans* biofilm supports this. A number of variables may be associated with detachment of microbial cells from a biofilm (*29*), resulting in erosion or sloughing. Flushing, which could mechanically shear the biofilm, could result in detachment of cells or aggregates that could in turn colonize the bloodstream and cause signs and symptoms of bacteremia. This phenomenon has been recently reported (*28*).

The case-patients in this outbreak had their CVCs removed and were treated with antimicrobial agents. The presence of *A. xylosoxidans* biofilm and the mechanism of bloodstream infection after disruption by catheter flushing suggests that eradication of infection would require catheter removal, as reported by others (*4*,*9*). Previously, recurrent *A. xylosoxidans* bacteremia has been reported in those patients whose indwelling catheters were not removed (*11*). Formation of *A. xylosoxidans* biofilm provides an explanation for the organism most commonly causing bacteremia in patients with CVCs (*7*,*10*).

The California Code of Regulations, Title 17, Section 2500 (*30*), requires all healthcare professionals to immediately report outbreaks of any cause; however, this outbreak was not recognized early on. The initial cluster of patients at Office B had symptoms and positive blood cultures growing this uncommon organism for 6 weeks before the cases were reported to the Los Angeles County Department of Public Health. Because outpatient settings may lack surveillance systems, outbreaks may not be recognized immediately, thus potentially exposing more patients. In addition, some of the symptoms of bloodstream infection were initially attributed to side effects of chemotherapy. Because 10% of patients in our prospective cohort study had blood cultures positive for *A. xylosoxidans*, further studies are needed to determine whether active surveillance for patients with CVCs would help recognize infections.

Because we noted not only infection control breaches but also that unlicensed office personnel were manipulating the CVCs, line flushes, infusions, and blood collection through CVCs, we reported the situation to the California Medical Board and the California Department of Health Services. Although no California state regulations for infection control in outpatient physician’s offices exist, the California Department of Health Services and Los Angeles County Department of Public Health recommended that the oncology office improve infection control standards; handling, storage, exposure, and access to pharmaceuticals; and improve medical record documentation. New infection control policies were established, and the office subsequently hired new, properly licensed registered nurses and nurse practitioners to handle insertion of intravenous catheters and administration of intravenous medications and chemotherapy. After proper education of the oncology office staff and removal of multidose vials of heparin and saline, no more *A. xylosoxidans* bloodstream infections were reported from Office B.

Our investigation has limitations. We did not culture *A. xylosoxidans* from the multidose vials. The original vials, used when the outbreak began, had already been discarded and were not available for testing by the time we were notified in January 2002. Our investigation was also limited by the absence of medical records indicating when nursing staff accessed the CVCs. Although the contamination of multidose vials remains suggestive, we suspect that they were the most likely source. The outbreak ended after discontinuing their use and enacting improved infection control practices. The organism could have been introduced into multidose vials by a needle or syringe used on an infected patient or by the artificial fingernails of the nurse, through gaps in infection control.

For patients who received infusion therapy at home, receipt of therapy in an outpatient clinic or physician’s office was an independent risk factor for bloodstream infection (*31*). Therefore, clinicians need to be vigilant because minor breaches in infection control can lead to large outbreaks with uncommon human pathogens, especially in patients with CVCs. Clinicians also need to ensure that appropriate infection control practices are adhered to consistently, especially in outpatient care settings, where oversight of infection control procedures may be absent. Unlike standards that exist for nursing homes or hospitals, no written standards regarding infection control in outpatient settings exist from the California Department of Health Services or the California Medical Board. However, routine monitoring and adherence to infection control could prevent outbreaks. Clinicians providing care in outpatient settings should review appropriate infection control standards and consider establishing written policies to ensure that standards are met. As healthcare delivery continues to move toward outpatient care (*32*), the lack of formal infection control procedures and accountability in the outpatient office setting can lead to large disease outbreaks (*33*,*34*); the need for oversight in this setting should be considered.

Our investigation helps characterize the mechanisms of *A. xylosoxidans* bloodstream infection in immunocompromised patients with CVCs. It provides a better understanding of how biofilm formation in a CVC can result in a clinical infectious disease process with this uncommon organism. Substantial illness and death can occur in outpatient settings that lack formal oversight. This outbreak highlights an unaddressed infection control problem in the outpatient setting for regulating agencies to further review.
